# Machine learning-based classification of circadian rhythm characteristics for mild cognitive impairment in the elderly

**DOI:** 10.3389/fpubh.2022.1036886

**Published:** 2022-10-28

**Authors:** Zhizhen Liu, Lin Zhang, Jingsong Wu, Zhicheng Zheng, Jiahui Gao, Yongsheng Lin, Yinghua Liu, Haihua Xu, Yongjin Zhou

**Affiliations:** ^1^National-Local Joint Engineering Research Center of Rehabilitation Medicine Technology, Fujian University of Traditional Chinese Medicine, Fuzhou, China; ^2^College of Rehabilitation Medicine, Fujian University of Traditional Chinese Medicine, Fuzhou, China; ^3^School of Biomedical Engineering, Health Science Center, Shenzhen University, Shenzhen, China; ^4^Marshall Laboratory of Biomedical Engineering, Shenzhen University, Shenzhen, China

**Keywords:** mild cognitive impairment, circadian rhythm, wrist-wearable sensors, ecological transient assessment, machine learning

## Abstract

**Introduction:**

Using wrist-wearable sensors to ecological transient assessment may provide a more valid assessment of physical activity, sedentary time, sleep and circadian rhythm than self-reported questionnaires, but has not been used widely to study the association with mild cognitive impairment and their characteristics.

**Methods:**

31 normal cognitive ability participants and 68 MCI participants were monitored with tri-axial accelerometer and nocturnal photo volumetric pulse wave signals for 14 days. Two machine learning algorithms: gradient boosting decision tree and eXtreme gradient boosting were constructed using data on daytime physical activity, sedentary time and nighttime physiological functions, including heart rate, heart rate variability, respiratory rate and oxygen saturation, combined with subjective scale features. The accuracy, precision, recall, F1 value, and AUC of the different models are compared, and the training and model effectiveness are validated by the subject-based leave-one-out method.

**Results:**

The low physical activity state was higher in the MCI group than in the cognitively normal group between 8:00 and 11:00 (*P* < 0.05), the daily rhythm trend of the high physical activity state was generally lower in the MCI group than in the cognitively normal group (*P* < 0.05). The peak rhythms in the sedentary state appeared at 12:00–15:00 and 20:00. The peak rhythms of rMSSD, HRV high frequency output power, and HRV low frequency output power in the 6h HRV parameters at night in the MCI group disappeared at 3:00 a.m., and the amplitude of fluctuations decreased; the amplitude of fluctuations of LHratio nocturnal rhythm increased and the phase was disturbed; the oxygen saturation was between 90 and 95% and less than 90% were increased in all time periods (*P* < 0.05). The F1 value of the two machine learning algorithms for MCI classification of multi-feature data combined with subjective scales were XGBoost (78.02) and GBDT (84.04).

**Conclusion:**

By collecting PSQI Scale data combined with circadian rhythm characteristics monitored by wrist-wearable sensors, we are able to construct XGBoost and GBDT machine learning models with good discrimination, thus providing an early warning solution for identifying family and community members with high risk of MCI.

## Introduction

Sleep disorder, especially circadian rhythm disturbance, is a common form of mild cognitive impairment (MCI) among adults and is a high risk factor for progression to dementia (1, 2). However, the impairment of sleep can be subjectively exaggerated in patients with MCI (3, 4). Therefore, evidence from objective measures is essential for constructing a more comprehensive model to understand the influence of the relative amplitude of the circadian rhythm of sleep and wake on the onset of MCI. Wearable technologies allow objective, ecologically transient long-term monitoring of physiology and behavior for motion tracking as well as sleep and circadian rhythm assessment (5). These physiological and behavioral assessments achieve minimal interference and are able to detect subtle changes in specific parameters. They are being increasingly used in clinical studies of chronic disease in the community, with particular appeal in dementia. A recent systematic assessment of the use of wearable technology (6) showed a decrease in daily activity levels as well as sleep efficiency and an increase in circadian rhythm variability in patients with dementia.

However, data from similar MCI studies with wearable technology become complicated due to variations in study objectives, procedures, feature extraction, and statistical methods, particularly for estimating the mean and variability of circadian rhythms (7). Moreover, few studies have been able to simultaneously estimate three key indicators of circadian activity (e.g., rhythm amplitude, timing, and variability) while also considering changes in circadian rhythms over multiple days in the same subject.

Therefore, we further studied the objective evidence of continuous circadian rhythm monitoring for early detection of community-dwelling older adults with MCI, and explored whether MCI classification model with good diagnostic efficiency could be constructed after adding the evidence of objective circadian rhythm measurement. Participants were assessed on the MoCA and PSQI scales and completed a 14-day triaxial accelerometer and day-time physical activity and night-time photoplethysmographic (PPG) signal recording with simultaneous sleep diary recording. Records of daytime physical activity and nocturnal photoelectric capacitance pulse wave signals were extracted to analyze the association of daytime physical activity (PA), sedentary time, and rhythmic amplitudes of nocturnal physiological functions with MCI.

## Methods

### Participants

We recruited 68 elderly individuals with MCI living in the community, and 31 normal cognition participants were selected and matched for age and sex.

#### Inclusion criteria

(1) Patients were eligible for participation if they had been diagnosed with MCI according to the 2018 Chinese Guidelines for the Diagnosis and Treatment of Dementia and Cognitive Impairment (8); (2) aged between 60 and 75 years old; (3) had normal activities of daily living, self-care or basic self-care; (4) understood and cooperated, participated and signed the informed consent form voluntarily.

#### Exclusion criteria

(1) Geriatric Depression Scale (GDS-15) score>8 (9), or a history of depression; (2) brain tumors, Parkinson's disease, unstable other medical conditions that can affect brain function or influence the evaluation of cognitive function; (3) history of acute illness within 3 months; (4) current diagnosis of active epilepsy; (5) secondary sleep-wake rhythm disorder due to physical illness or mental disorder; (6) participation in other clinical trials that could affect the evaluation of the results of this study.

Ethical approvals were granted by Ethics Committee of the Rehabilitation Hospital affiliated to Fujian University of Traditional Chinese Medicine (2019KY-002-02) and Ethics Committee of the Second People's Hospital of Fujian Province (SPHFJP-K2019001-1). We also registered this study in the Chinese Clinical Trial Registry (ChiCTR-ICR-15005795). All participants provided informed consent before the initiation of study procedures.

### Evaluation projects

#### General demographic information

Basic demographics, behavioral lifestyle, health status and other factors were assessed using basic information questionnaires.

#### Cognitive function assessment

(1) Montreal Cognitive Assessment (MoCA)

The Fuzhou version of the MoCA scale was used to assess the overall cognitive function of the subjects face-to-face. The preliminary study of the project team proved that the Fuzhou version of the MoCA scale has good reliability and structural validity with satisfactory factor loadings on the corresponding factors (10), including eight cognitive domains such as executive function, visuospatial structure, memory, attention, verbal fluency, abstraction, calculation and orientation. The total MoCA score was 0–30 (a higher score equates to better function), with ≥26 being normal, between 18 and 26 being mild cognitive impairment, between 10 and 17 being moderate, and less than 10 being severe.

(2) Ascertain Dementia 8-item Questionnaire (AD8)

The AD8 Dementia Screening Scale was developed by the University of Washington in 2005 and it contains 8 items (11). The Chinese version of the scale uses a score of ≥2 as the cutoff value for cognitive impairment, with a sensitivity of 85.7% and a specificity of 77.6% (12). Because the Chinese version of the AD8 is less time-consuming and is easy for older adults to understand and self-assess, it has good potential for widespread use in community and non-specialized medical settings such as general medicine.

(3) Instrumental Activities of Daily Living Scale (IADLs)

Developed by Lawton et al. with good reliability and validity (13). The scale contains eight entries on telephone use, shopping, food preparation, household maintenance, laundry, transportation, medication management, and financial management, with a total score of 0 to 23, with higher scores representing more complete activities of daily living abilities. A score of less than 2 standard deviations from the norm means that the ability to perform activities of daily living is severely impaired (14).

(4) Geriatric Depression Scale-15 (GDS-15)

Simplified from Burke et al. (15), the Chinese version of the scale has an internal consistency Cronbach's α coefficient of 0.82 (16). The scale contains 15 items, of which 1, 5, 7, 11, and 13 are reverse scored, and the remaining 10 are positive scored, each with a score of 0 or 1, giving a possible high score of 15. The higher the score is, the more pronounced the depressive tendency. A GDS-15 score >8 indicates the presence of depressive symptoms.

#### Subjective sleep quality assessment

The participants completed a recent 1-month sleep quality assessment using the internationally recognized Pittsburgh Sleep Quality Index scale (PSQI) (17). The Chinese version has been tested for reliability and validity and is suitable for assessing sleep quality in the Chinese population (18). The PSQI scale consists of 19 self-assessment items that comprise 7 dimensions, namely, sleep quality, time to sleep, sleep duration, sleep efficiency, sleep disorders, hypnotic drugs, and daytime dysfunction. Each dimension is scored on a 0–3 scale, and the cumulative score for each dimension is the total PSQI score, with a total score range of higher scores indicating a worse quality of sleep. A score of 0 to 5 is specified: sleep quality very well; scores >5 indicate a significant sleep disorder.

#### Objective circadian rhythm monitoring and feature extraction

**Figure 1:**
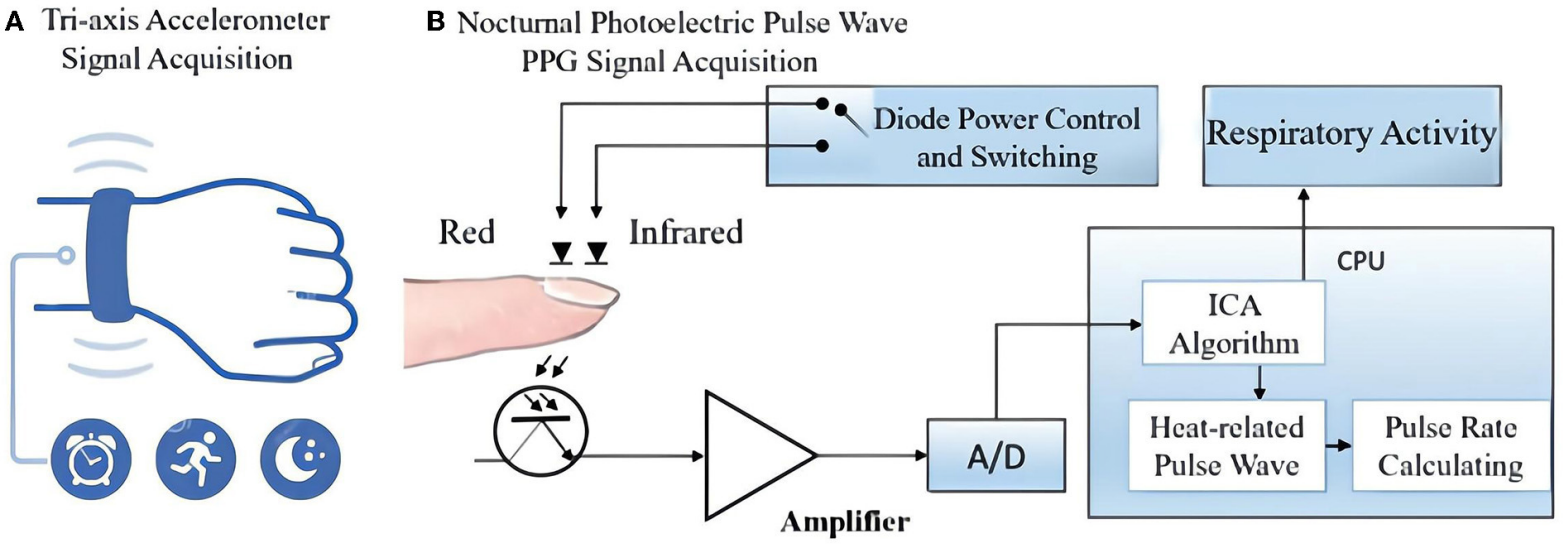
System setup: The data collection and analysis of wrist-wearable sensors. **(A)** The built-in tri-axial accelerometer records the acceleration signal of the wrist and converts it into a digital signal for storage; **(B)** two channels of transmission mode PPG signals were collected from the participants' index finger and analyzed using an independent component analysis (ICA) algorithm. The respiratory activity was separated from the heart-related pulsation in PPG after the ICA analysis.

This study used a wristwatch with a tri-axial accelerometer and an optical heart rate sensor (W180, Shenzhen Fitfaith Technology Co., Ltd.) as the data collection device (Figure 1). The accelerometer signals are sampled at 25 Hz, and the PPG signals are sampled at 100 Hz. This wristwatch is powered by a built-in battery, which has a maximum 12-hour usage time and supports USB charging.

Each subject was allocated two wristwatches for rotation. They were asked to wear the device at all times for a period of 14 days and not to remove it except for bathing, swimming, and rotating the watch. During the sleep period, subjects were required to attach a separate finger sleeve to the watch and wrap the sleeve around their index finger. The watch automatically activates an optical heart rate sensor using PPG. This sensor emits infrared light onto the skin to capture the pulse wave signal, which can measure physiological parameters such as heart rate and respiratory activity. Outside of this process, only the three-axis acceleration sensor in the watch works, recording the acceleration signal from the wrist. Each subject was also asked to record the time of going to bed and waking up in the morning each day in a sleep diary during the experiment.

The experiment was conducted in batches between September 2020 and March 2021. The 14-day experiment was conducted entirely on the subjects' own, without any intervention by the experimenter during this period. However, at the end of each batch, the experimenter will make improvements to the subsequent batches based on participant feedback and the quality of the collected data. Improvements include giving subjects more training before the experiment, upgrading the battery capacity of the wristwatch, providing interchangeable watch straps to avoid allergies, etc.

All data processing and statistical analysis steps are achieved on the MATLAB 2020b, The Mathwork, Inc. The implementation of the machine learning and the visualization of results are based on python toolkits such as scikit-learn and matplotlib.

(1) Data cleansing

Based on the examination of the experimental data, especially from the early batches, we found the following bad data: (1) the sensor of the subject's watch was damaged due to water and other special circumstances, resulting in abnormalities in the recorded acceleration and PPG signals and failure to present normal waveforms; (2) the subject did not charge the watch in time while wearing the watch, making the watch automatically shut down and unable to record rhythmic information; (3) the subject removed the watch due to the subject removed the watch because of discomfort, housework, etc. In this case, the watch recorded a null signal. These bad data were screened out by manual selection. According to the sleep logs filled out by the participants, more than 90% of the participants had a subjective sleep time before 0:00 am and a subjective wake time after 6:00 am, and most of them woke up in the middle of the night during this period. Therefore, the time period from 0:00 to 6:00 am was used as the entry window for the analysis of physiological nocturnal rhythms. Most of the participants woke up before 8:00 am and started their daytime activities, and the earliest time to sleep was after 8:00 pm, so the period from 8:00 am to 8:00 pm was used as the entry window for the analysis of the physical activity daily rhythm. If a piece of data for a subject is not included in this analysis time period, that data will be screened out.

(2) Data pre-processing

The wearable data acquisition device used in this experiment has a built-in filtering module, and the analog signal collected by the sensor is passed through the filtering module and the analog-to-digital conversion module to obtain the three-axis acceleration data and PPG data. The data were further filtered in order to filter out any possible interference during signal acquisition. For daytime triaxial acceleration data, a fourth-order Butterworth bandpass filter (0.2 Hz to 15 Hz) was used to remove the effects of body motion (19). For nighttime PPG data, since the PPG signal data recorded during sleep are affected by environmental disturbances and body movements, the PPG signal processing was performed by ensemble empirical mode decomposition (EEMD) with reference to the method of Motin et al. (20). We randomly added 100 groups of white noise to the original signal and extracted the scale components that fell within the range of physiological parameters such as heart rate and respiration of the elderly to obtain the denoised data.

(3) Daytime physical activity rhythm features

Indeed, there is some evidence that regular PA can favorably affect dementia patients' physical and cognitive function, quality of life, and activities of daily living (21). Most studies have used the ActiGraph as the gold standard tool for acquiring PA. Other studies have also used other acceleration monitoring devices as replacements (22). In this study, the sum of Count values was calculated from the acceleration data in 1-minute segments to calculate the PA. Based on the PA value, sedentary as well as the low-, medium-, and high-intensity activity (6, 23) can be classified. Specifically, the vertical axis acceleration signal was resampled to 30 Hz, and the acceleration values within a certain range were converted to the corresponding Count values in a sequence of 30 sample points and according to a threshold setting (24). A period of data longer than 90 min with PA kept at 0 was detected as a non-wear time and screened out (23). The analysis indicators and definition criteria of daytime PA are detailed in Table 1.

**Table 1:** Analysis indicators and definition criteria of daytime physical activity information.

**Analysis of indicators**	**Meaning**
Physical Activity (PA)	Sum of Count values in one minute
Sedentary	Moments when the PA value within the group was at 0–40%
Low Physical Activity	Moments when the PA value within the group was at 40%−70%
Median Physical Activity	Moments when the PA value within the group was at 70%−90%
High Physical Activity	Moments when the PA value within the group was at 90%−100%

Independent sample *t*-test to compare the differences in indicators between groups at each time period, with the significance level α set at 0.05.

(4) Nighttime physiological rhythm features

The PPG signal can be used to extract heart rate (HR), respiratory rate (RR), and oxygen saturation (SpO_2_) (25–27). The calculation module for these three physiological indices is already built into the wristwatch, so they can be extracted directly from the data. Heart rate variability (HRV) can be replaced by pulse rate variability (PRV) (28). The PRV-related indicators rMSSD, LF, HF, and LF/HF can be obtained from the PPG data (29). We divided the data into 30-second windows and extracted the average physiological indicators HR, RR, SpO_2_, rMSSD, LF, HF, and LF/HF from them (The formula to calculate the indicators can be seen in Table 2). After normalizing each type of feature by the Z-score method, the feature values obtained in all windows were concatenated into a long time series. It is also worth noting that the SpO_2_ data were also used to calculate the number of oxygen decreases obtained under each hour to estimate the quality of the subject's breathing during sleep. The duration of the oxygen desaturation (OD) events was also recorded as an analyzable point, where each oxygen desaturation event was defined in a way as a continuous decrease of more than 4% under one period (30). The percentage of time in each hour when SpO_2_ levels exceeded 95, 90–95%, and below 90% was used for analysis as well.

**Table 2:** Indicators of nocturnal physiological function and calculation formula.

**Indicators**	**Meaning**	**MATLAB formula**
rMSSD	Root mean square of the difference between adjacent pulse wave crest spacings	RMD [diff (PPi)]
LF	0.04–0.15 Hz in the pulse wave frequency band average power	Band power (wave, fs, [0.04, 0.15])
HF	0.15–0.4 Hz in the pulse wave frequency band average power	Band power (wave, fs, [0.15, 0.4])
LF/HF	Ratio of LF to HF	LF/HF
HR	Mean heart rate within a slice	Mean (HRs)
RR	Mean respiration rate within a slice	Mean (RRs)
SpO_2_	Mean peripheral oxygen saturation within the slice	Mean (SpO2s)
ODI	Number of oxygen desaturation in one hour	Length (ODs)
OD duration	The mean duration of oxygen desaturation in one hour	Mean (OD durations)
SpO_2_ ≥ 95%	Percentage of SpO_2_ content greater than 95%	Length [find (SpO_2_s >= 95)]/length (SpO_2_)*100
SpO_2_ 90–95%	Percentage of SpO_2_ content between 90 and 95%	Length [find (SpO_2_s 95|SpO_2_s >= 90)]/ length (SpO_2_)*100
SpO_2_ ≤ 90%	Percentage of SpO_2_ content less than 90%	Length [find (SpO_2_s = 90)])/ length (SpO_2_)*100

PPi is the wave spacing; fs is the sampling frequency of the signal; XXs is the data of the corresponding indicator within a segment; OD, oxygen desaturation.

The daytime and nighttime physical activity rhythm features of the MCI group and the cognitive normal group were averaged for each time period and presented as line graphs. To show the overall pattern and filter out the fluctuating data points, the median value was taken for each adjacent 5 min data, and 720 data points (i.e., the number of the data within the six-hour period) were compressed into 144 points. Moreover, the compressed data were further divided to four time periods. The average value of every feature was calculated.

#### Machine learning algorithm for MCI classification with multi-features training

(1) Feature extraction

To further investigate the relationship between nocturnal physiological rhythm features and MCI, this experiment was conducted by extracting the nocturnal rhythm features of the subjects and constructing machine learning models for the classification task of MCI. Specifically, we extracted HR, RR, SpO_2_, rMSSD, LF, HF, and LF/HF ratios between 00:00 and 06:00 for each group of data. With a window length of 20 min and a sliding window of 10-min stride, the median values of the data in each window were extracted. This results in 36 features for each group of rhythmic signals. In addition, we also extracted the average values of ODI, OD duration, SpO_2_ ≥95, SpO_2_ ≥90%, and the whole night of these indicators for each hour as a feature group, respectively. In total, a total of 8 feature groups with a total of 280 features were extracted.

(2) Feature selection

We use each feature group separately to train the random forest model. Each training group was performed using stratified ten-fold cross-validation. Also, because the number of samples in the MCI and control groups is different, stratified sampling is used to ensure that the sample distribution in the ten folds is the same. After the model training, the permutation importance was calculated ten times randomly on the test set (31). The results of the ten-fold cross-validation were finally averaged to obtain the importance of each feature within the feature group. The five most important features in each group were selected. Based on the median feature importance of the selected features, only the features with higher feature importance were retained for the subsequent classification task.

(3) MCI classification model

We empirically found that Gradient Boosting Decision Tree (GBDT) (32) and eXtreme Gradient Boosting (XGBoost) (33) performed best on this dataset compared to other machine learning models. Therefore, they were used for the classification task of MCI. Grid search (34) was applied to find the best hyper-parameters for both models. The list of hyper-parameters includes the number of base learners, the learning rate, and the maximum depth of the tree structure.

Compared with cross-validation, leave-one-out can fully use each data and get a more comprehensive performance evaluation. However, for the dataset in the study, each subject may have more than one data. There is correlation among the data belong to one subject. Therefore, leave-one-out cannot be used in a conventional way because of the risk of data leakage (35). Instead, we did leave-one-out on the subject list, i.e., each time the data from one of the subjects would be the test set, while the other data would be the training set. It not only solves the data leakage problem but also make highly use of the data.

We used the subject-based leave-one-out for grid search and evaluating the performance of the best model. The area under the ROC (AUC) was adopted to find the best hyper-parameters of the grid search. In addition to AUC, Accuracy, Precision, Recall, and F1-score were regarded as the evaluation metrics of the best model. ROC of the classification result was used to evaluate the performance as well. Besides, both GBDT and XGBoost are ensemble learning models, allowing the measurement of the feature importance through the calculation of the information gain brought by each feature under each base learner. We present the feature importance in a bar chart, where the height of the most important feature is set to 100, and the height of other features is adjusted accordingly.

To check whether subjective scale features can contribute to the performance of the MCI classification model, we added the subjective scale features that have significant differences between the MCI group and the control group to the dataset and trained new models. Furthermore, we used the performance of the models trained from data with exclusive subjective scale features for comparison. It is to be noted that, in this case, only one data was used for each subject instead of the same amount of data as the records the subject has.

## Results

### Research subject characteristics

Compared with cognitively normal controls, the MCI group had a lower level of education, a higher proportion of sleep disorder, and 2 or more chronic diseases (*P* < 0.05), as shown in Table 3. PSQI scores and their subdomain entries sleep disturbances, daytime dysfunction had intergroup differences, as detailed in Table 4.

**Table 3:** Comparison of clinical characteristics between the two groups who completed wrist-wearable sensors monitoring (*n* = 99).

**Variables**	**Mild cognitive impairment**		**Z/χ^2^**	***P*-value**
	***N*o *n* = 31**	**Yes *n* = 68**		
Female *n* (%)	17 (54.8)	43 (63.2)	0.629	0.428
Age, years M (IQR)	66 (6)	67 (9)	−0.544	0.586
Education, years M (IQR)	12 (7)	9 (4)	−2.692	0.007
SBP (mmHg) M (IQR)	128 (10)	126 (15)	−0.225	0.822
DBP (mmHg) M (IQR)	80 (15)	80 (9)	−0.460	0.646
BMI (Kg/m^2^) M (IQR)	23.7 (4.4)	23.7 (3.4)	−0.485	0.628
GDS-15 M (IQR)	1 (2)	3 (3)	21.924	0.020
Sleep Disorders *n* (%)	11 (35.5)	39 (57.4)	4.407	0.044
MoCA M (IQR)	27 (4)	22 (4)	−18.309	< 0.001
2 Chronic Diseases and Above *n* (%)	6 (19.4)	29 (42.6)	5.055	0.025

SBP, systolic blood pressure; DBP, diastolic blood pressure; BMI, body mass index; BMI = weight (in kg)/height^2^ (in m^2^); GDS-15, geriatric depression scale-15; MoCA, montreal cognitive assessment; Sleep disorders, PSQI total score ≥ 6.

**Table 4:** Comparison of Pittsburgh sleep quality scale scores between the two groups (*n* = 99).

**PSQI component**	**Mild cognitive impairment**		***z***	***P*-value**
	**No *n* = 31**	**Yes *n* = 68**		
Sleep quality	1.10 (0.65)	1.30 (0.87)	−1.276	0.206
Sleep latency	0.94 (0.85)	0.96 (0.84)	−0.107	0.915
Sleep duration	0.97 (0.84)	1.24 (1.13)	−1.329	0.188
Sleep efficiency	0.61 (0.96)	1.04 (1.24)	−1.890	0.063
Sleep disturbances	1.06 (0.36)	1.27 (0.59)	−2.105	0.038
Sleeping medication	0.13 (0.56)	0.15 (0.58)	−0.161	0.872
Daytime dysfunction	0.65 (0.66)	1.22 (1.03)	−3.351	0.001
Global PSQI score	5.42 (2.78)	7.18 (4.40)	−2.398	0.019

### Physical activity day rhythm characteristics

**Figure 2:**
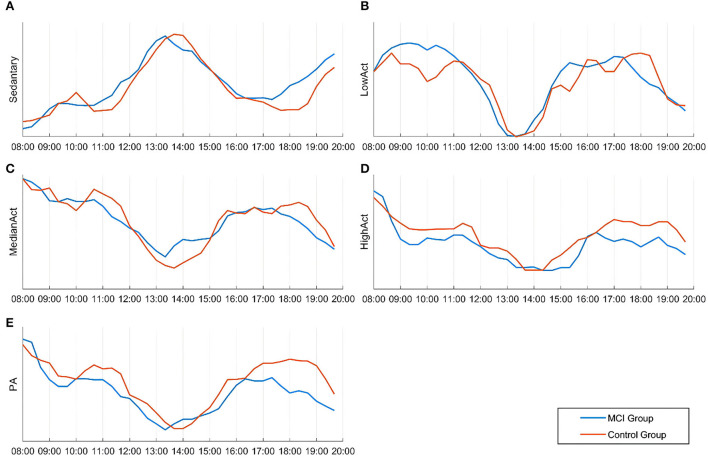
Differences in the temporal distribution of activity intensity and the amount of activity in various states between the two groups during 08:00–20:00. **(A)** Sedentary state; **(B)** Low physical activity state; **(C)** Median physical activity state; **(D)** High physical activity state; **(E)** Physical activity.

As shown in Figure 2, the amplitude rhythm fluctuations of sedentary, low physical activity, median physical activity, and high physical activity remained essentially the same between 8:00 and 20:00 for participants in the MCI and cognitively normal groups. The sedentary state basically reaches its peak between 12:00 and 15:00, when most elderly people are in a state of rest after eating, mainly sitting and lying in bed, and the amount of activity is greatly reduced. In the evening, the MCI group was more sedentary than the cognitively normal group between 17:00 and 20:00 and this reached another peak at approximately 20:00 (a).

As shown (e) in Figure 2, the trend of daily rhythmic variation of PA intensity in both groups of participants was highest from 8:00 to 9:00, lowest throughout the day from 13:00 to 14:00, and rebounded to some extent from 17:00 to 19:00. Compared to the cognitively normal group, the MCI group showed a significant decrease in PA intensity between 11:00 and 12:00, and the same occurred between 17:00 and 19:00. For the daily rhythm trend of the medium physical activity state (c), no statistically significant differences were found between groups; the daily rhythm trend in the low physical activity state was higher in the MCI group than in the cognitively normal group between 8:00 and 11:00 (b) and the daily rhythm trend in the high physical activity state was generally lower in the MCI group than in the cognitively normal group (d).

As shown in Table 5, the MCI group had lower activity levels than the normal cognitive group in all six time periods except “13:00–15:00” after normalizing the data, and the difference was statistically significant (*P* < 0.05). During 13:00–15:00, participants in both groups were generally resting after lunchtime and their activity level reached its daytime trough; the difference between the groups was not statistically significant (*P* > 0.05).

**Table 5:** Comparison of daytime activity between groups for each time period.

**Time**	**Mild cognitive impairment**	
	**No**	**Yes**
08:00–09:00	0.805 ± 0.084	0.752 ± 0.157^a^
09:00–11:00	0.593 ± 0.082	0.529 ± 0.061^a^
11:00–13:00	0.455 ± 0.152	0.351 ± 0.124^a^
13:00–15:00	0.165 ± 0.072	0.172 ± 0.077
15:00–17:00	0.497 ± 0.116	0.446 ± 0.128^a^
17:00–19:00	0.676 ± 0.083	0.473 ± 0.08^a^
19:00–20:00	0.381 ± 0.146	0.282 ± 0.074^a^

^a^Indicates compared to the cognitively normal group, *P* < 0.05.

### Physiological function night rhythm characteristics

**Figure 3:**
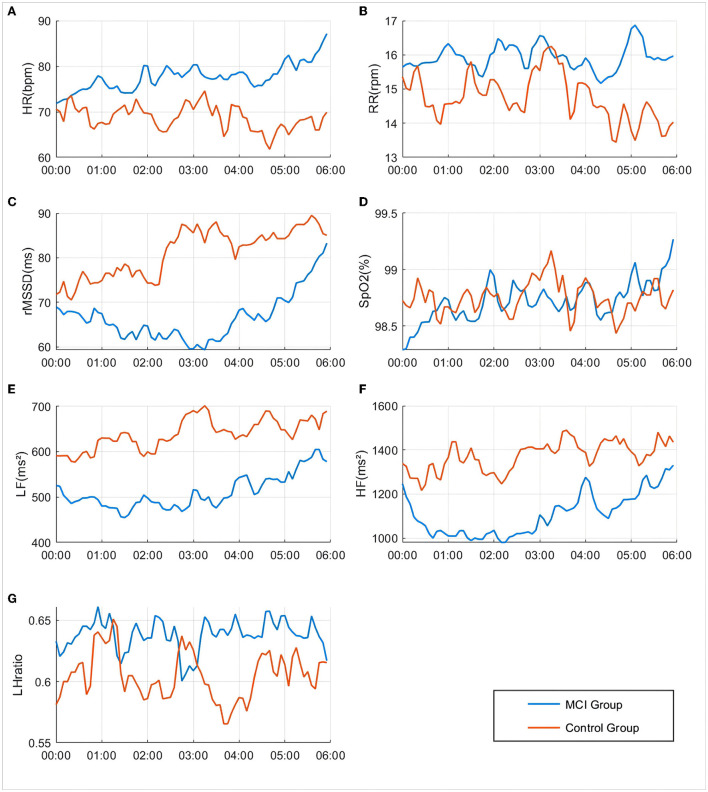
Differences in changes in various physiological indicators between groups during 00:00–06:00. **(A)** HR: Heart rate; **(B)** RR: Respiratory rate; **(C)** rMMSD: Root mean square of the difference between adjacent pulse wave crest spacings; **(D)** SpO_2_: Peripheral oxygen saturation; **(E)** LF: Low Frequency, 0.04–0.15 Hz in the pulse wave frequency band average power; **(F)** HF: High Frequency, 0.15–0.4 Hz in the pulse wave frequency band average power; **(G)** LHratio: Ratio of LF–HF.

As seen in Figure 3, the rMSSD-HRV, LF-HRV, and HF-HRV in the cognitively normal group of older adults showed a nocturnal elevated rhythm, peaking at approximately 3:00 a.m. Compared with cognitively normal older adults, the peak rhythm of the rMSSD-HRV, LF-HRV, and HF-HRV at 3:00 a.m. disappeared, and the amplitude of fluctuations decreased in the MCI group. Elevated amplitude and phase disturbance of the nocturnal rhythm fluctuations of the low-frequency to high-frequency component ratio (LHratio), a parameter reflecting sympathetic-parasympathetic interactions, indicates that the autonomic stability of MCI patients is impaired, and their relative activity of sympathetic and parasympathetic nerves is out of balance. In addition, the amplitude of nocturnal rhythm fluctuations in heart rate and respiration was elevated in the MCI group, and the rhythm range was essentially the same.

**Figure 4:**
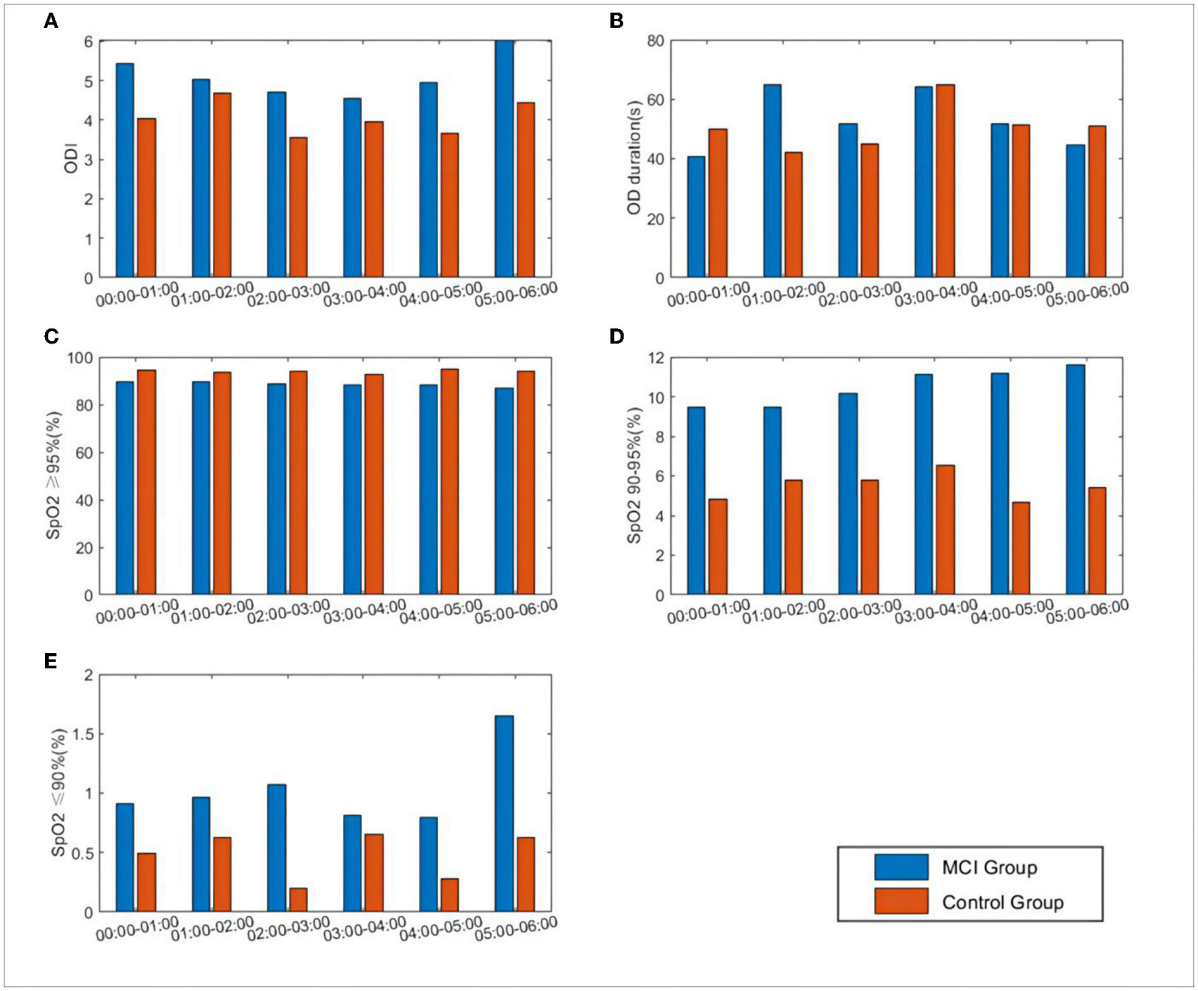
Differences in SpO_2_ and related oxygen desaturation indexes between the two groups during 0:00–6:00. **(A)** ODI: oxygen desaturation indexes, number of oxygen desaturation in one hour; **(B)** OD duration: the mean duration of oxygen desaturation in one hour; **(C)** SpO_2_ = 95%: percentage of SpO_2_ content greater than 95%; **(D)** SpO_2_ 90–95%: percentage of SpO_2_ content between 90 and 95%; **(E)** SpO_2_ = 9%: percentage of SpO_2_ content less than 90%.

The mean oxygen desaturation duration of the MCI patients at 1:00–3:00 was elevated compared to that of patients with normal cognitive function (*P* < 0.05). The percentage of pulse oxygen saturation (SpO_2_) in the range of 90–95% and less than 90% was elevated throughout the night from 0:00 to 6:00 (*P* < 0.05). There was no statistically significant difference in the comparison of ODI and SpO_2_ greater than 95% (*P* > 0.05), as shown in Figure 4.

For HR, RR, SpO_2_, rMSSD, LF, HF, and the LHratio, the median window was expanded to 10 min to extract the features, and for ODI, OD duration, SpO_2_ ≥95%, and SpO_2_ 90–95% we extracted the values in each hour as features and calculated them separately from the total mean value for each time period. As shown in Table 6, the nocturnal oxygen desaturation index was reduced in MCI patients, consistent with the range of rhythmic changes in oxygen saturation and HRV time-domain/frequency-domain indices.

**Table 6:** Distribution of each index of physiological function between groups in each time period.

**Time**	**00:00–01:00**		**01:00–03:00**		**03:00–05:00**		**05:00–06:00**	
**Indicators**	**MCI**	**Control**	**MCI**	**Control**	**MCI**	**Control**	**MCI**	**Control**
HR	74.8 ± 58.5	69.4 ± 42.8	77 ± 65.3	69.2 ± 40.8	77.7 ± 68	68.3 ± 36.6	83 ± 74.3	67.6 ± 32.2*
RR	15.8 ± 11.4	14.8 ± 8.5	16 ± 12.8	14.9 ± 7.8	15.8 ± 13.2	14.9 ± 7.4	16.2 ± 14.4	14.1 ± 6.1*
SpO_2_	98.5 ± 4.2	98.7 ± 2.8	98.7 ± 4.6	98.7 ± 2.7	98.7 ± 4.7	98.8 ± 2.4	99 ± 4.9	98.8 ± 2.1
rMSSD	67.3 ± 64.7	73.9 ± 40.6	63.1 ± 55.8	79 ± 48.6*	65.1 ± 50.6	84.4 ± 51.8*	76.7 ± 62.8	87.4 ± 53.2
LF	497.7 ± 352.6	594.8 ± 358.1*	480.1 ± 339.4	629.7 ± 367.8*	519.8 ± 359.6	663.3 ± 368.1*	578.7 ± 389.2	669.2 ± 360*
HF	1072.9 ± 1153.3	1297.6 ± 1112.6	1015 ± 993.3	1355.1 ± 1025.9*	1150.9 ± 1258.2	1424.4 ± 965*	1257.9 ± 1180.8	1411.4 ± 931.1
LF/HF	0.6 ± 0.2	0.6 ± 0.2	0.6 ± 0.2	0.6 ± 0.2	0.6 ± 0.2	0.6 ± 0.2*	0.6 ± 0.2	0.6 ± 0.2
ODI	5.4 ± 7	4 ± 6.6*	4.9 ± 5.6	4.1 ± 4	4.7 ± 4.8	3.8 ± 3.5*	6 ± 6.6	4.4 ± 4.8*
OD duration	40.8 ± 45.3	50.1 ± 77.9	58.2 ± 90.9	43.6 ± 68.1	57.9 ± 100	58.1 ± 58.9	44.6 ± 44	51 ± 80.4
SpO_2_ ≥95%	89.6 ± 17.9	94.7 ± 13*	89.2 ± 18.3	93.8 ± 13.9*	88 ± 19.7	93.9 ± 12.1*	86.8 ± 21.9	94 ± 12.1*
SpO_2_ 90–95%	9.5 ± 16.4	4.9 ± 12*	9.8 ± 16.2	5.8 ± 13.4*	11.2 ± 18.4	5.6 ± 11.3*	11.6 ± 19.3	5.4 ± 11.3*
SpO_2_ ≤ 90%	0.9 ± 5.8	0.5 ± 2.4	1 ± 6.5	0.4 ± 2.1	0.8 ± 6.1	0.5 ± 1.9	1.7 ± 9.3	0.6 ± 3.4

^*^The MCI group and the cognitively normal (Control) group with statistically significant differences for that index and for that time period.

HR, heart rate; RR, respiratory rate; SpO_2_, peripheral oxygen saturation; LF, low frequency, 0.04–0.15 Hz in the pulse wave frequency band average power; HF, high frequency, 0.15–0.4Hz in the pulse wave frequency band average power; LF/HF, ratio of LF to HF; ODI, oxygen desaturation indexes, number of oxygen desaturation in one hour; OD, duration, the mean duration of oxygen desaturation in one hour; SpO_2_ ≥95%, percentage of SpO_2_ content greater than 95%; SpO_2_ 90–95%, percentage of SpO_2_ content between 90 and 95%; SpO_2_ ≤ 9%, percentage of SpO_2_ content less than 90%.

### Classification performance of MCI classification models

After data cleansing, only 436 data records were used for training MCI classification models. Among them, 293 records were collected from 49 MCI group subjects, while 143 records were collected from 24 control group subjects. We first performed feature selection on the extracted features based on the permutation importance calculated from the random forest. Table 7 presents the importance of the five most important features in each group of features. Among the eight group of the features, rMSSD and HF have more important feature (higher than median level); HR and SpO_2_ performs relatively worse, where only two features are important; RR, LF and LF/HF has no features contribute to the classification of MCI. In general, 20 important features were selected for training the MCI classification model, which greatly reduced the training burden of the models.

**Table 7:** The top five most important features of each feature group.

**OD**	**HR**	**RR**	**SpO_2_**	**rMSSD**	**LF**	**HF**	**LF/HF**
04:00–05:00*	00:20–00:40*	00:10–00:30*	02:10–02:30*	03:40–04:00*	03:40–04:00	00:00–00:10*	02:40–03:00*
00:00–01:00*	01:10–01:30*	02:50–03:10	02:30–02:50*	02:40–03:00*	03:50–04:10	01:50–02:10*	03:50–04:10
03:00–04:00	00:00–00:10*	00:30–00:50	02:20–02:40	00:00–00:20*	05:20–05:40	02:30–02:50*	05:10–05:30
Mean SpO_2_ 90–95%	03:10–03:30*	00:00–00:10	00:40–01:00	00:10–00:30*	01:40–02:00	05:10–05:30*	02:10–02:30
SpO_2_ 90–95% 03:00–04:00	00:40–01:00	00:00–00:20	00:00–00:20	02:50–03:10*	00:10–00:30	03:30–03:50*	03:30–03:50

^*^The importance of the features with bold mark are higher than median level.

We used the grid search to tune the hyper-parameters of the models. For both GBDT and XGBoost, the same hyper-parameters combination grid was used, including the number of base learners, the learning rate, and the maximum depth of the tree structure. At last, comparing the average AUC of the subject-based leave-one-out, the above hyper-parameters were set as [150, 0.01, 3] for GBDT and [200, 0.05, 3] for XGBoost.

Table 8 shows the results of the best-performed MCI classification models based on GBDT and XGBoost. Besides, the performance of the model trained from the data added with subjective scale features and that contains only subjective scale data are also presented. The adopted subjective scale features include education (EDU), daytime dysfunction (DD), sleep disturbance (SD), PSQI score, and type of chronic disease (CD). As mentioned, the significant difference in these features between the MCI and control groups has been proved.

**Table 8:** The performance of the MCI classification models (units: %).

**Model**	**Training data**	**Accuracy**	**Precision**	**Recall**	**F1 score**	**AUC**
GBDT	P	66.74	69.89	88.74	78.20	62.00
	P+S	74.31	74.79	93.17	82.98	58.72
	S	75.69	75.20	95.22	84.04	62.83
XGBoost	P	69.27	73.73	84.30	78.66	65.10
	P+S	69.95	75.00	82.94	78.77	64.88
	S	69.50	75.64	80.55	78.02	70.68

P, features extracted from physiological function indicators; S, subjective scale features; XGBoost, eXtreme gradient boosting; GBDT, gradient boosting decision tree.

**Figure 5:**
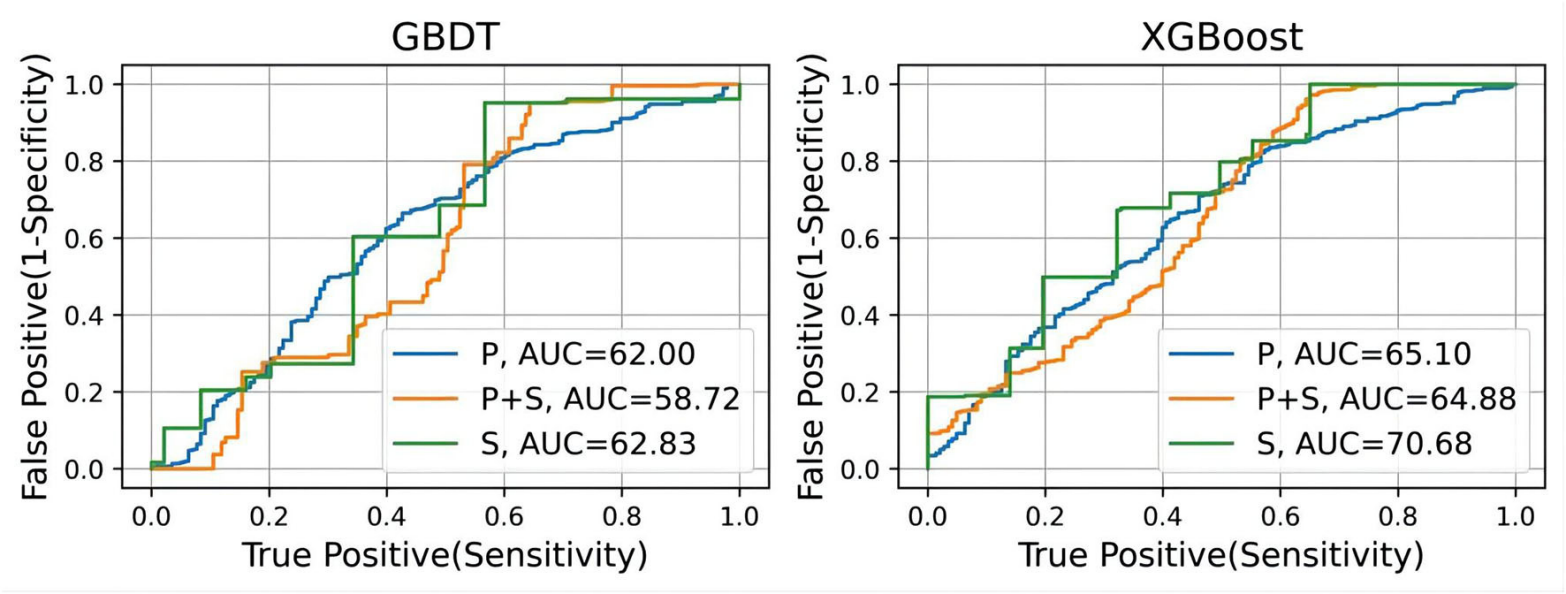
The ROC curves of the MCI classification model including GBDT and XGBoost. Receiver operating characteristic curves. AUC (C statistics) is the corresponding values of the area under the curve for each model.

As seen in the table, the model trained from exclusive subjective scale data performs best for both models, while the model trained from physiological function features performs worst. Combining these two types of features would improve the performance of the MCI classification. Moreover, GBDT performs much better than XGBoost except for the model without subjective scale features training. From another view, the results of the recall metric are approximately 20% higher than the precision metric. This difference indicates that the classification models can classify most MCI data. However, some normal cognitive subjects may be misclassified as MCI patients. F1 score is the trade-off between these two metrics. All the results are around 80%, which shows that the models have good classification performance. However, as for the AUC metric, all the results, especially GBDT models, are not impressive, which can be seen more clearly in the ROC curve (Figure 5).

**Figure 6:**
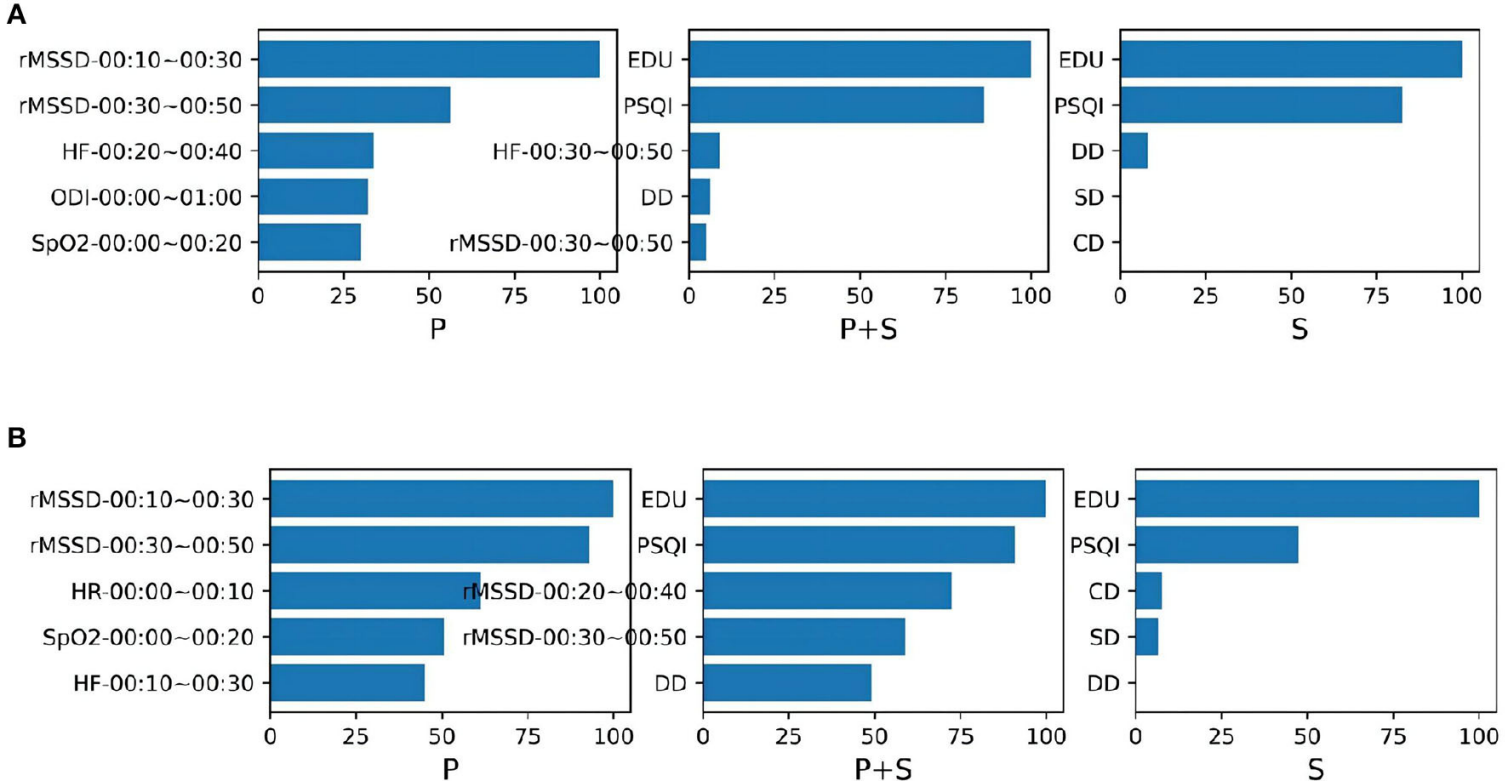
The top five importance features contribute to the MCI classification models **(A)** GBDT and **(B)** XGBoost. Both models were trained from the data containing the physiological function features (P), the data containing the subjective scale features (S), and the data of their combination (P + S).

We also presented the top five important features in each GBDT and XGBoost classification models (Figure 6). From the figure we can see that for the models trained from physiological function features, two rMSSD features are most important. Education and the PSQI score contribute greatly to the classification of the MCI no matter whether the training data contain physiological function. For the GBDT model trained from both type of data, the performance of the rMSSD features is quite close to these two subjective scale features. It is to be noted that all important physiological function features are extracted from the data in 00:00~01:00. This discovery may be helpful to further research on feature extraction.

## Discussion

This study compared indicators of awake and sleepy circadian rhythm patterns and describes more specifically the differences in rhythm characteristics between the groups. Some research questions are answered: (1) Wrist-wearable sensors are an effective means of monitoring the early cardiopulmonary coupling characteristics of MCI. Objective evidence of disturbances in the nocturnal rhythm of physiological functions is manifested by an imbalance in the relative activity of sympathetic and parasympathetic nerves and impaired stability of autonomic functions, particularly evident from 1:00 to 3:00. The daily amount of total physical activity and high physical activity decreases. The periods of 5:00–7:00, 7:00–9:00, and 17:00–19:00 may be the key time windows for “adapting measures according to time”. (2) Two optimal machine learning models (XGBoost and GBDT) were constructed using PSQI data combined with objective features of circadian rhythms monitored by wrist-wearable sensors, and they have a high accuracy and precision of classification and a good differentiation of MCI.

### Characteristics of changes in nighttime autonomic function in MCI

Sleep is a complex state characterized by important changes in the autonomic regulation of cardiovascular and respiratory activity. Abnormal autonomic regulation is highly associated with negative affect and sleep disturbances, especially during NREM sleep, possibly due to abnormal parasympathetic regulation of cardiac activity (36). HRV is an important indicator commonly used in clinical practice for the non-invasive detection of autonomic function. The results of this study found that the time domain index HRV and the frequency domain indices LF-HRV and HF-HRV had significantly lower rhythm amplitudes and higher LF/HF ratio rhythm amplitudes during sleep in MCI patients than in cognitively normal controls. This means that the autonomic function of MCI patients is impaired, the relative activity of sympathetic and parasympathetic nerves is imbalanced, and the body's ability to adapt to the environment is decreased. This is similar to the findings of other researchers (37). As a result, they are prone to clinical manifestations such as difficulty sleeping, easy waking, reduced concentration, and forgetfulness.

In recent years, several studies have also found that early cognitive decline may be associated with cardiac autonomic dysfunction. Nonogaki et al. (38) assessed cardiac autonomic function in AD patients by HRV and found that the memory domain of cognitive function in AD was closely related to cardiac autonomic function. Marte et al. (39) showed that compared to cognitively normal elderly people, MCI and AD patients had altered HRV indicators at 70° of postural tilt, mainly in the form of increased HF and decreased LF and LF/HF. The LF/HF ratio is an indicator of sympathetic activation, suggesting a poor sympathetic response to postural stress in patients with MCI and AD. A study published in 2020 in the journal Sleep (25) showed that patients with MCI had greater reductions in HF-HRV during NREM sleep than those with subjective cognitive decline, suggesting that HF-HRV may be an early biomarker for detecting dementia.

In addition, we also found important changes in the nocturnal rhythm of breathing in MCI patients, and the ODI and low oxygen ratio (SpO_2_ less than 90%) were higher than those in the cognitively normal group, especially during the 1:00–3:00 period. This is consistent with the results of similar studies. Kazuko et al. (40) found that a decrease in the apnea/hypopnea index (AHI) at night and SpO_2_ in elderly individuals had a negative impact on cognitive function. Severe nocturnal hypoxemia reflects impaired functional connectivity of medial temporal brain structures, which are involved in the pathophysiology of sleep, memory and dementia (41).

The association between respiratory and cardiac rhythms is widely recognized. Our study also confirmed that the rhythm ranges of nocturnal SpO_2_, ODI and HRV time/frequency domain indicators in MCI patients were consistent. This demonstrates that respiratory signal and HRV analysis is a useful tool for non-invasive and accurate monitoring of respiratory activity and autonomic regulation of cardiac activity and for assessing changes in cardiopulmonary coupling during sleep in patients with MCI.

### Characteristics of changes in daytime physical activity levels in MCI

Although current evidence (42) suggests that sleep fragmentation, daytime sleepiness, and circadian rhythm disturbances are common in AD, it is unclear how these factors influence MCI activity of daily living patterns, intensity and peak activity phase. Our study found two distinct peak phases in the 12 h physical activity daily rhythm (10:00–11:00, 16:00–17:00) and two distinct trough phases (13:00–14:00, 19:00–20:00) in older adults during the period 8:00–20:00. This suggests that short breaks after lunch become the norm for older adults. A cross-sectional study of 2,214 individuals living in several cities in China (43) showed that healthy older adults aged 60 years and older who took afternoon naps had significantly higher MMSE cognitive ability scores than the non-napping group, as well as significant differences in positional awareness, verbal fluency, and memory. Furthermore, a research team from the Institute of Chronic Diseases, affiliated with Zhejiang University School of Medicine, investigated the relationship between nap duration and different metabolism-related diseases in 3,327 people aged 18–80 years in four communities in Lanxi City (44), and the findings suggest that keeping afternoon naps to one hour may have potential benefits for the prevention of diabetes and its related diseases.

However, we did not find differences in the phases of total daytime physical activity amplitude between MCI and cognitively normal older adults during the 12-hour daytime rhythms. The MCI group was more sedentary than the cognitively normal group between 17:00 and 20:00. This finding is consistent with the evidence that people with AD can maintain many activities of daily living abilities in the preclinical phase (45) and suggests that early cognitive decline may not affect most daily physical activities. Despite having similar physical function and physical activity capacity, MCI patients exhibited a decrease in physical activity compared to controls. Available evidence suggests that sedentary behavior is associated with decreased cognitive and physical function (46) and that increased physical activity may moderately promote health benefits (47). It not only emphasizes the importance of daytime physical activity intensity affecting cognitive decline but also illustrates the potential value of early exercise rehabilitation for the MCI population before significant declines in physical activity and function (48).

When total physical activity was further divided into high, medium and low intensities to compare the differences between groups, it was found that the daily rhythm amplitude of the low physical activity state was higher in the MCI group than in the cognitively normal group from 8:00 to 11:00. Daily rhythm amplitude in the high physical activity state was generally lower in the MCI group than in the cognitively normal group. Medium physical activity status was basically the same in both groups. This suggests that MCI patients may have limited capacity for high physical activity. Previous studies (49) have shown that older adults prefer family-centered activities and that cognitive decline is associated with narrower living spaces and more time spent at home. MCI patients may spend less time in complex environments outside of the home, which may not be conducive to high-intensity physical activity. Many high-intensity physical activities (e.g., dancing, running, ball games) may be more physically demanding and cognitively complex than low- to moderate-intensity physical activities (e.g., cleaning, cooking, walking) and may be more challenging for individuals with MCI. These individuals may also be cognitively more dependent on caregivers or other adults to facilitate physical activity programs, such as during self-care or out-of-home visits. Therefore, individuals with MCI are an important target population for moderate-to-vigorous physical activity interventions that moderately increase daily physical activity and reduce sedentary time. Tsai et al. (50) found that aerobic and resistance exercise were effective in increasing neurotrophic proteins, reducing certain inflammatory cytokines, and promoting neurocognitive function in older adults with MCI. However, the two exercise modalities may promote cognitive function through different neuromolecular mechanisms. A study by Tao et al. (51) confirmed the potential of traditional Chinese medicine exercise for MCI as represented by Baduanjin. The Baduanjin group showed increased gray matter volume in the right hippocampal region compared to the walking group and increased resting-state functional connectivity between the hippocampus and the right angular gyrus in the Baduanjin group compared to the health education group. Future studies should consider more multimodal interventions for motor rehabilitation programs in the MCI population.

### Advantages and applications of machine learning techniques

The use of wearable watch devices has made it possible to capture physiological functional signals during sleep. Subjects wore a finger cover equipped with an optical heart rate sensor during sleep, whereby their real-time PPG signal could be measured. We extracted nocturnal physiological functional characteristics from the PPG signals. After data filtering, feature extraction, and feature screening, GBDT and XGBoost are constructed to classify prediction of MCI. We validate the classification performance of the model by the subject-based leave-one-out method. From the results, both models have great MCI classification performance. The results of recall metric are much higher than that of precision, which indicates that although the model can classify most MCI subjects, there is a portion of the control group who are misclassified. However, this is not bad news, because MCI can be ruled out for false-positive patients by going through more standard MCI diagnostic procedures. The high F1 score also shows that the MCI classification models with nocturnal physiological features has some potential for MCI diagnosis. Nonetheless, we can see from the ROC curve that the model does not perform well on the AUC metric, which indicates that the classification results are quite dependent on the threshold setting.

We also compared the models trained with the addition of subjective scale features and with only these features each. These features have been shown to be significantly different in the MCI and control groups. From the results, it appears that the inclusion of these subjective scale features can effectively improve the classification ability of the models. In fact, the models trained by these features alone already have good classification results. But by real-time physiological function signals, especially like heart rate variability features rMSSD as well as HF, belong to ecological transient assessment. Compared with the traditional assessment model, this ecological transient assessment model, which can provide real-time feedback of circadian rhythm changes in the natural state, is suitable for long-term monitoring at home and in the community and can well compensate for the waste of limited medical resources.

The advantage of the machine learning model in this study is that it can improve the decision-making ability of older adults and their caregivers. First, machine learning models can integrate higher-order nonlinear interactions between predictor variables and outcomes (52). Thus, our model can accommodate large databases and can also identify interactions between features. Machine learning can be easily recalibrated over time for model iterations as new data become available for subsequent studies (53), forming ecological transient solutions for MCI alerts. In addition, advanced machine learning models have the advantage of being scalable because they can update models by automatically interfacing with community or hospital EHR data and integrating digital images, natural language processing, and continuous monitoring of physiological data (54). Therefore, machine learning, as a very important assistive technology for continuous monitoring and early warning of MCI, can further improve the triage decision-making ability of health care providers to identify people at high risk of MCI among the community elderly.

However, it cannot be denied that this study was limited by the sample size, and external validation of the model performance is something that needs to be further explored. Future iterations of the new model could be used to support decision-making for MCI diagnosis and prediction of referral to constitute a comprehensive data-based machine learning decision support system for health care professionals in the community or health care facilities. In addition, the classification model can be developed for self-assessment of traditional Chinese medicine constitution and PSQI scales by older adults at home, combined with wrist-wearable sensor monitoring data to decide whether to visit a medical facility for a professional neuropsychological assessment.

### Shortcomings and prospects

This study benefited from the use of a large sample of community-dwelling older adults from our prior cross-sectional study in Fuzhou, China. MCI participants were diagnosed by a physician. All participants wore wrist-wearable sensors for at least 3 days or more in a free-living environment, PPG signals were captured at night, and 24-hour objective monitoring data were combined with subjective scale assessment data. The results of this study provide potential evidence for the subsequent design of targeted rehabilitation interventions.

There are several limitations in this study. First, the use of a wrist-worn accelerometer did not record certain specific physical activities, including swimming, cycling, and lower body activities, and therefore may have underestimated total physical activity. Second, the study was conducted in the Fuzhou community without an external validation dataset. It may not be generalizable to other cities. Future longitudinal follow-up and mechanistic studies based on this work will help us better understand how circadian rhythm disturbances in wakefulness and sleep affect cognitive decline and brain pathological changes associated with MCI.

## Conclusion

Patients with MCI have impaired autonomic function stability, imbalance in the relative activity of sympathetic and parasympathetic nerves, disturbance in the amplitude of nocturnal rhythm fluctuations of HRV and ODI, a decrease in total daytime physical activity and high physical activity, and decreased adaptability of the body to the environment. By collecting subjective PSQI scale data combined with objective circadian rhythm features to construct MCI machine learning classification models with good discriminatory power, ecologically transient solutions for MCI early warning are formed that may be useful in cognitive monitoring and care of older adults at home and in the community.

## Data availability statement

The raw data supporting the conclusions of this article will be made available by the authors, without undue reservation.

## Ethics statement

Ethical approvals were granted by Ethics Committee of the Rehabilitation Hospital affiliated to Fujian University of Traditional Chinese Medicine (2019KY-002-02) and Ethics Committee of the Second People's Hospital of Fujian Province (SPHFJP-K2019001-1). The patients/participants provided their written informed consent to participate in this study.

## Author contributions

ZL conceptualized the study, analyzed the findings, and wrote the manuscript. ZL, LZ, and JG searched the literature, selected studies, and extracted the data. ZL, ZZ, YLin, JW, YLiu, and HX contributed to the analysis, interpretation of the data, and provided important scientific input. YZ and ZL supervised the whole study. All authors collaboratively discussed key decisions throughout the course of the review, provided critical feedback on preliminary manuscript, and approved the final version.

## Funding

This study is funded by the National Natural Science Foundation of China (Grant No. 81973928) and the Natural Science Foundation of Fujian Province, China (Grant Nos. 2020L3013 and 2022J06028).

## Conflict of interest

The authors declare that the research was conducted in the absence of any commercial or financial relationships that could be construed as a potential conflict of interest.

## Publisher's note

All claims expressed in this article are solely those of the authors and do not necessarily represent those of their affiliated organizations, or those of the publisher, the editors and the reviewers. Any product that may be evaluated in this article, or claim that may be made by its manufacturer, is not guaranteed or endorsed by the publisher.
